# Insights into *Pneumocystis jirovecii* pneumonia in South Africa (2018–2022)

**DOI:** 10.1093/mmy/myaf001

**Published:** 2025-01-23

**Authors:** Mbali Precious Nkosi, Keegan John Hoog, Michelle Lowe

**Affiliations:** Department of Clinical Microbiology and Infectious Diseases, University of the Witwatersrand, Johannesburg, South Africa; Ineos Oxford Institute for Antimicrobial Research, University of Oxford, Oxford, UK; Department of Clinical Microbiology and Infectious Diseases, University of the Witwatersrand, Johannesburg, South Africa

**Keywords:** *Pneumocystis jirovecii* pneumonia, South Africa, proportion, epidemiology, retrospective study

## Abstract

*Pneumocystis jirovecii* causes *P. jirovecii* pneumonia (PJP)—a leading opportunistic infection among persons with advanced human immunodeficiency virus. Furthermore, individuals with underlying conditions such as cancer and transplant recipients are susceptible to PJP. Most data on PJP come from other countries, with limited knowledge about its prevalence in Africa. The aim was to describe changes in the proportion of positive PJP tests in South Africa from 2018 to 2022. A 5-year retrospective study was conducted on patients with suspected *P. jirovecii*, detected by polymerase chain reaction. Data were obtained from the National Health Laboratory Service, where laboratory diagnostics were done as part of routine patient care. Mann–Whitney test and *Χ*^2^ tests were used to compare the age, sex, and wards with both the negative and positive results of PJP. From 2018 to 2022, a total of 8110 patients’ results were retrieved, and 8059 met the inclusion criteria. The positive test proportions of PJP in South Africa were 32.66%, 29.93%, 34.02%, 24.98%, and 25.78%, respectively. The median age was 35 years, with interquartile range of 24–43 years (*P* = .002). Female patients had a higher positive test proportion than males (59.39% vs. 38.74%, *P* < .001). The proportion of positive PJP tests was higher in general wards (48.54%) and intensive care units (18.99%) (*P* = .012). The epidemiology of PJP in South Africa is similar to that of other countries in some respects but is influenced by unique factors specific to the country. These findings are crucial for public health planning, emphasising the need for targeted PJP prevention strategies considering sex- and age-specific vulnerabilities in South Africa.

## Introduction

*Pneumocystis jirovecii*, formerly known as *P. carinii*, was first identified in underweight and preterm newborns in the middle of the 20th century.^1^ Due to the rise in the occurrence of *P. jirovecii* pneumonia (PJP) during the Human Immunodeficiency Virus/Acquired Immunodeficiency Syndrome (HIV/AIDS) epidemic in the 1980s, it became uniquely associated with HIV/AIDS.^2,3^ However, with the widespread availability of antiretroviral medication in many countries, individuals living with HIV/AIDS are now less likely to be infected with PJP.^4^

Currently, there is an increase in PJP rates among individuals who do not have HIV/AIDS but have other conditions that compromise the immune system, such as cancer, solid organ or bone marrow transplants, or the use of anti-inflammatory medications like corticosteroids.^5^*Pneumocystis jirovecii* severely affects the health of those it infected, causing serious symptoms, such as breathing difficulties, fever, dry cough, and in severe cases, respiratory failure, which can be fatal.^2,6,7^ As a response to the alarming morbidity and mortality rates of life-threatening PJP in immunocompromised individuals, the World Health Organization has listed *P. jirovecii* as one of the 19 priority fungal infections.^8^ Reported mortality rates are as high as 65% in individuals with moderate disease and as high as 84% in those with respiratory failure requiring ventilation and admission to the intensive care unit (ICU).^5^ Individuals who have this infection require prompt and efficient treatment.^3^

The current state of PJP in sub-Saharan Africa, particularly in South Africa, is not well documented. Current data frequently lack comprehensive national estimates and are either out-of-date or population-specific.^9^ To properly estimate the present burden and guide public health interventions, updated epidemiological data on the proportion of PJP in all at-risk categories are crucial. Addressing this gap is essential to adjusting preventative and treatment plans, particularly as the number of immunocompromised people keeps increasing.

## Aim

This study aimed to use retrospective data to record and investigate changes in the proportion of positive PJP tests in South Africa over a period of 5 years from 2018 to 2022.

## Methods

### Study design and setting

A retrospective study was conducted at the University of the Witwatersrand, Department of Clinical Microbiology and Infectious Diseases. Ethical approval was obtained from the University of the Witwatersrand Human Research Ethics Committee (ref. no.: M230415).

### Study population

The study population consists of patients admitted to South African government hospitals between 2018 and 2022 who were suspected of having PJP based on normal diagnostic procedures, which can include, but are not limited to, laboratory, radiographic, or significant indicative clinical signs. To aid in diagnosis, these patients underwent polymerase chain reaction (PCR) testing for *P. jirovecii* unless the sample was received in a broken container or if it was a nasopharyngeal or oropharyngeal swab. Demographic data, including age, sex, and ward of admission, were collected to assess the proportion of positive PJP tests across different subgroups. The population spans a wide age range, from infants to the elderly, and includes patients from various hospital wards, such as general wards and ICUs.

### Criteria for suspecting PJP infection and initiating sample testing

Suspicion of PJP infection was based on a combination of risk factors, clinical features, and radiological findings. Risk factors included underlying immune suppression, such as HIV with CD4 counts below 200 (a common scenario in South Africa); patients who had undergone solid organ transplants and were on immunosuppressants, patients with haematological malignancies, preterm infants, and patients receiving chronic disease-modifying agents or biologics. These patients are at a high risk of PJP infection.

Clinically, the infection often presented with classic signs of respiratory distress, including low blood oxygen saturation and difficulty breathing. Radiologically, the hallmark finding was a diffuse ground-glass appearance in both lung fields, although variability could occur depending on the disease stage. Additional testing, such as β-D-glucan (BDG) assays, typically showed markedly elevated levels in cases of PJP. Combining these factors increases the likelihood (pre-test probability) of a positive result and informs the decision to proceed with PCR testing.

### Data extraction and analysis

Patient data were retrieved from the PJP molecular database maintained by Infection Control Services (ICS), National Health Laboratory Service (NHLS). Yearly proportions were defined as the number of positive *P. jirovecii* cases over the total number of tested cases in a specific year. Provincial proportions were calculated as the total number of positive cases in each province over the total number of positive cases from 2018 to 2022. Eligible patients included those admitted to government hospitals in South Africa from 2018 to 2022, suspected to be infected with *P. jirovecii*. Patients who had a positive *P. jirovecii* result but were missing age, sex, or ward information were included; however, 51 patients’ data were excluded from analysis as they were external quality control samples and not patient samples. Analysed data included the age, sex, ward, and *P. jirovecii* results. The retrieved hospital data were, therefore, categorised into their respective provinces to see which province had the highest proportion of positive PJP tests in South Africa. All wards, such as surgical, admission, ICU, paediatrics, maternity wards, and general wards, were analysed, and from that information, we were able to see which wards had a higher positive test proportion. The age of patients informed us which age group is more at risk for contracting PJP. Analysing sex helped us in observing whether males or females had a higher proportion of positive PJP tests. The proportion of positive PJP tests was calculated using the following formula:


\begin{eqnarray*}
{\mathrm{proportion}} = \frac{{\mathrm{positive}}\,\,{{P.\,\,jirovecii}}\,\,{\text{samples}}}{\mathrm{Total\,\,number\,\,of\,\,tested\,\,samples}}\times 100.
\end{eqnarray*}

Microsoft Excel® 2016 (Microsoft Corp., USA) data software was used for data cleaning, sorting, and filtering. All statistical tests were done using the Stata statistical software package, version 17 (StataCorp, 2021). Age was the only continuous variable and was not normally distributed; hence, it was represented as the median and interquartile range (IQR). Sex and wards were categorical variables and were represented as numbers (percentages). Mann–Whitney test was used to assess the statistical significance between age and the PJP result, and a *Χ*^2^ test was used to compare whether the result of PJP was associated with sex and ward. The *P*-value was set at *P* = .05, and any value whereby, *P* < .05, was considered statistically significant.

### Molecular tests

Molecular tests were not performed in this study, but were performed by ICS, NHLS, as part of routine patient care. In brief, DNA extraction was conducted using one of two methods: manual extraction with the QIAamp DNA Mini Kit (Qiagen, Germany) or automated extraction with the Magna Pure LC total nucleic acid isolation kit (Roche Diagnostics Corp., USA) on the MagNA Pure instrument (Roche Diagnostics Corp., USA). The extracted DNA was used immediately for real-time PCR or stored at −10°C to −25°C. Real-time PCR, targeting the major surface glycoprotein gene and including an internal control, was conducted using the LightCycler 480 instrument (Roche, USA).

## Results

### Description of the study population

During the 5-year period of this study, a total of 8110 isolates were tested by the ICS, NHLS, of which 8059 met the inclusion criteria and were included in the final analysis. The majority of the specimens were received from Gauteng (69.08%; *n* = 5567/8059) followed by KwaZulu-Natal (11.74%; *n* = 946/8059), North West (7.23%; *n* = 583/8059), Eastern Cape (4.43%; *n* = 357/8059), Western Cape (2.27%; *n* = 183/8059), Limpopo (2.21%; *n* = 178/8059), Free State (1.39%; *n* = 112/8059), Mpumalanga (1.28%; *n* = 103/8059), and the Northern Cape (0.37%; *n* = 30/8059).

### PJP yearly and provincial positive test proportions

**Figure 1.**
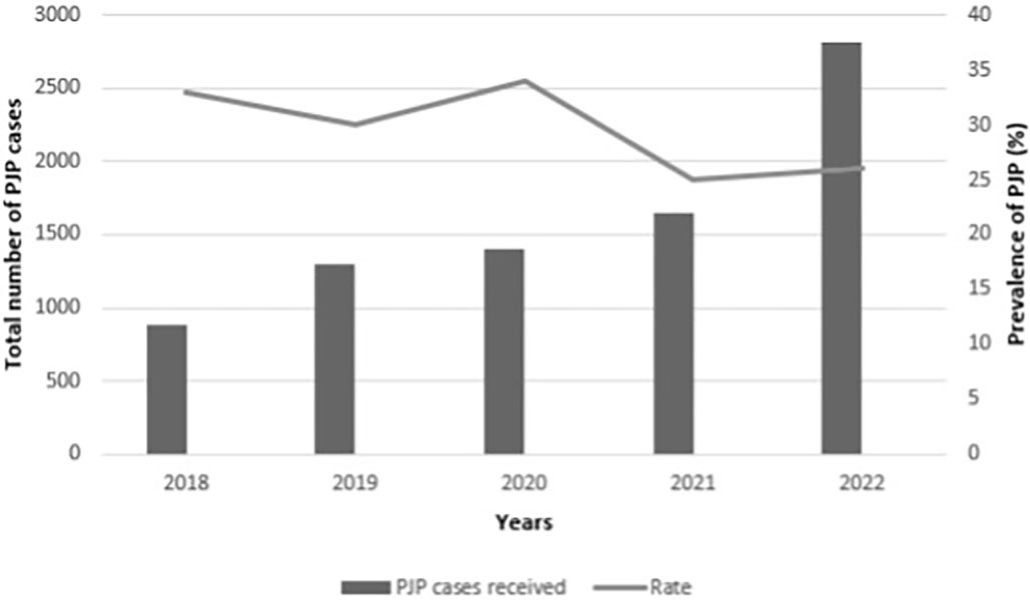
Proportions of positive PJP tests in South Africa from 2018 to 2022 (*n* = 8059). The bar chart illustrates the total number of suspected PJP cases (both positive and negative) across the years 2018–2022, while the line graph shows the positive PJP test rate during the same period. The left *y*-axis represents the total number of suspected PJP cases, peaking in 2022 with over 2500 cases. The right *y*-axis displays the positive PJP test proportion rate in percentage, with the highest rate observed in 2020 coinciding with the COVID-19 pandemic, followed by a decline in 2021 and stabilisation in 2022.

**Figure 2.**
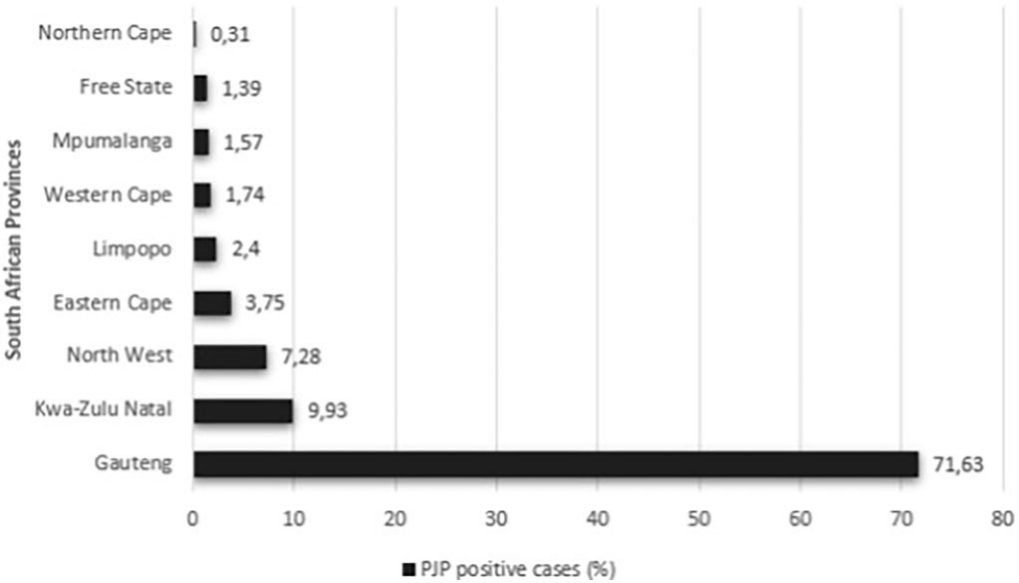
Total number of positive PJP cases in each province in South Africa, 2018–2022 (*n* = 2295). The horizontal bar chart shows the distribution of PJP positive cases as a percentage across the nine provinces of South Africa between 2018 and 2022. Gauteng accounts for the highest proportion of PJP cases, with 71.63% of the total, followed by KwaZulu-Natal at 9.93%. The provinces with the lowest percentages are Northern Cape (0.31%), Free State (1.39%), and Mpumalanga (1.57%). This figure highlights significant provincial disparities in the occurrence of PJP, with a concentration of cases in Gauteng due to the high population density.

The proportion of positive PJP tests among patients who underwent testing from 2018 to 2022 was 32.66% (*n* = 281/891), 29.93% (*n* = 387/1293), 34.02% (*n* = 477/1402), 24.98% (*n* = 413/1653), and 25.78% (*n* = 727/2820), respectively (Fig. 1). Provinces with higher proportions were Gauteng (71.63%; *n* = 1644/2295), KwaZulu-Natal (9.93%; *n* = 228/2295), and North West (7.28%; *n* = 167/2295), while the other provinces had a positive test proportion of less than 5% (Fig. 2).

### Clinical characteristics of patients with PJP

Among all patients, 28.48% (2295/8059) tested positive for PJP by PCR. The majority of patients in this study were female (59.39%; 1363/2295) (Table 1). The highest number of cases occurred in the 31-to-40-year-old group (28.76%, 660/2295), followed by children in the 0-to-10-year-old group (19.35%, 444/2295) from 2018 to 2022. General wards constituted approximately half of all positive PJP cases (48.54%; 1114/2295), and there was a higher proportion of children patients admitted to ICU (39.41%; 175/444) than adults (15.31%; 261/1705).

**Table 1.** All patients who tested positive for PJP reported to ICS, NHLS, 2018–2022.

Characteristics	Patients reported, *n* (%)
Sex (*n* = 2295)	
Male	889 (38.74)
Female	1363 (59.39)
Unknown	43 (1.87)
Age group (*n* = 2295)	
Infants and children (0–10 years)	444 (19.35)
Young adults (11–20 years)	40 (1.74)
Adults (21–59 years)	1592 (69.37)
Older adults (>60 years)	73 (3.18)
Unknown	146 (6.36)
Wards patients were admitted in (*n* = 2295)	
General wards	1114 (48.54)
Surgical wards	7 (0.31)
Female wards	132 (5.75)
Male wards	68 (2.96)
Paediatric wards/ICU	138 (6.01)
Neonatal ICU	37 (1.61)
Adults ICU	261 (11.37)
Medical wards^⁋^	118 (5.14)
Accident, emergency, and trauma	83 (3.62)
Infectious disease wards^†^	124 (5.40)
Other wards^§^	149 (6.49)
Unknown	64 (2.79)

ICS = Infection Control Services; NHLS = National Health Laboratory Service; ICU = intensive care unit.

⁋ Medical OPD, medical emergency, medical casualty, N4B medical ward, acute medical unit, medical ward A, medical endocrinology/cardiac.

† ARV clinic, TB units, infectious diseases, isolation ward, virology clinic, MDR TB ward, House Idahlia COVID unit, gastro/haematology/oncology, endocrinology/respiratory/GI/metabolic ward, haematology oncology, rheumatology/nephrology, cardiology.

§ 7B2 extension ward, high care unit, step-down ward a and b, short stay ward, physician on call, yellowwood, adult burns unit, folateng ward, empilweni clinic, outpatient department, casualty.

## Discussion

Herein, we conducted a retrospective study to generate yearly and provincial positive test proportion for PJP by analysing data from ICS, NHLS, which included government hospitals from nine South African provinces. We examined patients’ age, sex, and the ward they had been admitted to. Our study indicates several interesting features, such as a higher proportion of PJP positive tests in female patients, a concentration of cases among children between the ages of 0 to 10 years, and adults between the ages of 31 to 40 years, and a considerable presence of PJP in general wards.

The proportion of positive PJP tests from 2018 to 2022 was 32.66%, 29.93%, 34.02%, 24.98%, and 25.78%, respectively. Gauteng had a higher proportion of PJP positive tests compared to other provinces, which may be attributed to its high population density.^10^ Additionally, Gauteng is home to some of the largest and most comprehensive healthcare facilities in South Africa, offering extensive diagnostic resources for immunocompromised patients, including those with HIV/AIDS or other conditions requiring regular testing.^11^ This increased testing capacity in Gauteng may result in a higher detection rate of PJP cases compared to provinces with fewer diagnostic resources. Studies have shown a prevalence of 24% among patients with suspected PJP, with an additional 17% detected through PCR in cases with negative microscopy results.^12^ These findings are comparable to the results of our study.

The variation in sampling size over the study period could result from fluctuations in healthcare demand. During certain periods, such as flu seasons or public health crises, more people visit hospitals, potentially leading to higher sample submissions. In other times, such as during summer or festive periods, fewer patients may seek care, resulting in fewer samples. Secondly, the impact of the Coronavirus Disease 2019 (COVID-19) pandemic drastically altered healthcare access and usage. During peak periods, hospitals prioritised COVID-19 cases, reducing the collection and testing of non-COVID-related samples. Lockdowns and travel restrictions may have also limited patient access to the hospital, leading to lower sample submissions in certain years. Lastly, changes in patient behaviour during the pandemic also played a role. Many patients avoided hospitals due to fear of infection, possibly reducing the number of non-COVID-related samples received. Conversely, during post-pandemic periods, there may have been a surge in patients returning for delayed healthcare needs, temporarily increasing sample submissions. There was a peak in 2020 (34.02%), during the COVID-19 outbreak; however, the rates decreased to approximately 25% in 2021 and 2022. These results are consistent with a study that investigated the effect of COVID-19 on the frequency of PJP.^13^ The authors reported a lower prevalence of PJP during the peak pandemic phase in South Africa, suggesting that the prevention strategies, such as isolation, mask usage, and travel restrictions, may have lowered exposure to *P. jirovecii* and subsequently the prevalence of PJP.^13^ The increase in proportion of positive tests in 2020 in South Africa could have been due to the increase of testing of all respiratory illness symptoms as a result of national guidelines implemented to counter the COVID-19 pandemic.^14^

According to our study, female patients have a higher proportion of positive PJP tests (59.39%). A *Χ*^2^ test was performed to compare whether the result of PJP was associated with sex, and the result showed statistical significance (*P* < .001) (Table 2). This result does not align with previous studies on PJP epidemiology, which frequently found higher rates of infection in male patients. Kolbrink et al. reported a higher prevalence among male patients with PJP, accounting for approximately two-thirds of their data (65.3%).^15^ Furthermore, Harizanov et al. conducted a prospective study and reported a predominance of male patients (81.3%) compared to female patients (18.7%).^16^ In contrast, male patients in this study account for slightly above one-third of our data (38.73%). Although the precise causes of this discrepancy have not yet been determined, a possible reason could be that in South Africa, most individuals who utilise the public healthcare system are mostly female,^17^ which could explain the higher proportion of positive PJP tests observed in our study.

**Table 2.** All patients with suspected PJP in South African patients, 2018–2022.

Variable		Negative	Positive	*P*-value
		*n* = (5649)	*n* = (2295)	
Sex	Male	2721 (48.97%)	889 (39.48%)	**<.001**
	Female	2836 (51.03%)	1363 (60.52%)	
Age		36 (21–47)	35 (24–43)	**.002**
Ward	Intensive care unit	154 (2.73%)	67 (2.92%)	**.012**
	Intensive care unit 4	396 (7.01%)	126 (5.49%)	
	Ward 15	137 (2.43%)	65 (2.83%)	
	Ward 20	633 (11.21%)	213 (9.28%)	
	Ward 4	163 (2.89%)	64 (2.79%)	
	Other	4166 (73.75%)	1760 (76.69%)	

Data are presented as median (IQR) for continuous measures, and number (percentage) for categorical measures.

There was a higher proportion of PJP positive tests in patients aged 31–40 years (28.76%). Patients aged 11–20 years (1.74%) and those older than 60 years (2.70%) had a lower proportion of positive PJP tests in South Africa. A Mann–Whitney test was performed to compare whether the result of PJP was associated with age, and the result showed statistical significance (*P* = .002) (Table 2). In contrast, Kolbrink et al. reported a higher prevalence among elderly patients aged 50–70 years, with a median patient age rangings from 62 to 64 years,^15^ and Maini et al. also reported a higher prevalence in elderly patients aged 60–69 years.^18^ However, Maini et al. could have missed the high prevalence in infants because their study was conducted during the period of HIV diagnosis, and they only included patients older than 15 years in their analysis.

The higher proportion of positive PJP tests in children aged 0–10 years may be related to factors such as immunological immaturity, underlying diseases, or environmental exposures.^19^ Individuals in the 31-to-40-year age group may be more vulnerable than others due to various factors. Firstly, individuals in this age group may be at a crossroads of several risk factors, including the incidence of HIV/AIDS, comorbidities, and exposure to medical environments. Further extensive epidemiological and clinical investigations are necessary to explore the particular causes of PJP in this age group.

Our study revealed a high proportion of PJP positive tests in general wards (48.54%). The second highest PJP positive test proportion was observed in the ICU ward (18.99%), which is consistent with findings from other studies.^13,20^ A *Χ*^2^ test was performed to determine whether the result of PJP was associated with the type of ward a patient was admitted to, and there was statistical significance (*P* = .012). This finding emphasises the significance of increased awareness and diagnostic inquiries for PJP in critically ill patients. Although PJP frequently affects immunocompromised individuals, the presence of PJP cases in the ICU underscores the severity of the condition and the critical care needs of these patients.

Our data do not specify whether PJP cases were hospital- or community-acquired. Patients in the ICU frequently have serious illnesses, and many may have weakened immune systems due to factors, such as invasive surgeries, organ dysfunction, and prolonged hospitalisation.^21^ These factors create an environment where opportunistic diseases, such as PJP, might flourish.

For early diagnosis and effective treatment, it is essential to understand the manifestation of PJP in the ICU. To improve patient outcomes, this environment should prioritise targeted prophylaxis, increased surveillance, and early detection of PJP symptoms.^22^ Although the ICU wards were the main focus of our study, it is important to recognise that a significant portion of PJP patients are also seen in general wards, which cover a wide range of patient care areas. Patients with less severe PJP signs are among the patients served by general wards. In order to offer a comprehensive picture of the disease’s effect and treatment outcomes, future research should examine the distribution and management of PJP across these various wards.

One limitation of this study is the absence of data on patient comorbidities, which restricts our ability to assess how underlying health conditions may have contributed to the observed high proportion of positive PJP tests. Information on comorbidities, particularly for immunocompromising conditions, could provide a deeper understanding of risk factors for PJP. Future studies incorporating detailed comorbidity data would enable a more comprehensive analysis of patient profiles and enhance the generalisability of these findings.

The epidemiology of PJP in South Africa is comparable to that in other countries in some respects, though there are variables unique to our country. This study showed higher proportions of positive PJP tests during the study period compared to other countries, particularly in female patients. Additionally, there was a higher proportion of positive PJP tests among young children (0–10 years) and adults (31–40 years), in contrast to the available literature. The findings in this study suggest that clinical practices in South Africa should develop targeted PJP prevention strategies that take into account sex-specific risk factors and age-specific vulnerabilities.
